# Exploring Fingerprints of the Extreme Thermoacidophile *Metallosphaera sedula* Grown on Synthetic Martian Regolith Materials as the Sole Energy Sources

**DOI:** 10.3389/fmicb.2017.01918

**Published:** 2017-10-09

**Authors:** Denise Kölbl, Marc Pignitter, Veronika Somoza, Mario P. Schimak, Oliver Strbak, Amir Blazevic, Tetyana Milojevic

**Affiliations:** ^1^Extremophiles Group, Department of Biophysical Chemistry, University of Vienna, Vienna, Austria; ^2^Department of Nutritional and Physiological Chemistry, Faculty of Chemistry, University of Vienna, Vienna, Austria; ^3^Department of Symbiosis, Max Planck Institute for Marine Microbiology, Bremen, Germany; ^4^Biomedical Center Martin, Jessenius Faculty of Medicine in Martin, Comenius University in Bratislava, Martin, Slovakia

**Keywords:** *Metallosphaera sedula*, Martian regolith simulants, EPR spectroscopy, microbe–mineral interactions, biosignatures

## Abstract

The biology of metal transforming microorganisms is of a fundamental and applied importance for our understanding of past and present biogeochemical processes on Earth and in the Universe. The extreme thermoacidophile *Metallosphaera sedula* is a metal mobilizing archaeon, which thrives in hot acid environments (optimal growth at 74°C and pH 2.0) and utilizes energy from the oxidation of reduced metal inorganic sources. These characteristics of *M. sedula* make it an ideal organism to further our knowledge of the biogeochemical processes of possible life on extraterrestrial planetary bodies. Exploring the viability and metal extraction capacity of *M. sedula* living on and interacting with synthetic extraterrestrial minerals, we show that *M. sedula* utilizes metals trapped in the Martian regolith simulants (JSC Mars 1A; P-MRS; S-MRS; MRS07/52) as the sole energy sources. The obtained set of microbiological and mineralogical data suggests that *M. sedula* actively colonizes synthetic Martian regolith materials and releases free soluble metals. The surface of bioprocessed Martian regolith simulants is analyzed for specific mineralogical fingerprints left upon *M. sedula* growth. The obtained results provide insights of biomining of extraterrestrial material as well as of the detection of biosignatures implementing in life search missions.

## Introduction

Chemolithoautotrophy has been indicated as the most primordial form of microbial metabolism on the early Earth (Blöchl et al., 1992; Wächtershäuser, 1992; Stetter, 2006) and proposed as a possible metabolic form for other iron-mineral-rich planets like Mars (Grotzinger et al., 2014; Hurowitz et al., 2017). Recent Mars exploration missions have provided a comprehensive analysis of the physical and geochemical environment of Mars (Grotzinger, 2013; Grotzinger et al., 2014; Hurowitz et al., 2017) aiming to identify potential habitats that bear the energy sources available on this planet to support chemolithotrophic life. The rich content of iron and sulfur minerals on Mars makes iron and sulfur transforming microorganisms the prime candidates for considering as models for putative Martian extant or extinct life forms (Amils et al., 2007; Nixon et al., 2012). In the hydrogeological and atmospheric oxygen-rich past of Mars (>3 Ga ago), such metabolically similar microorganisms might have contributed in redox cycling of elements from Martian regolith and deposition of the mineral sediments of hydrated sulfates and ferric oxide content (Amils et al., 2007). The detection of a stable redox-stratified water body along with recent mineralogical, geochemical and sedimentological investigations point to an ancient habitable fluvio-lacustrine environment at Yellowknife Bay in the Gale crater of Mars that would have been suited to harbor a Martian biosphere based on chemolithoautotrophy (Grotzinger, 2013; Grotzinger et al., 2014; Hurowitz et al., 2017). The present atmosphere of Mars contains traces of oxygen at a concentration of 0.146% (Mahaffy et al., 2013), ruling out the consideration of Mars as an environment where only anaerobic metabolism can be expected, while mineralogical analysis points to a plausible redox couple for prokaryotic respiration (Grotzinger, 2013; Grotzinger et al., 2014). In this context metal transforming extremophiles represent an exciting field of research for the study of microbe–mineral interactions in order to find the unique biosignatures of life in extreme conditions.

A rock-eating archaeon *Metallosphaera sedula*, originally isolated from a geothermal environment, flourishes in hot and acidic conditions (optimal growth at 74°C and pH 2.0) and exhibits unusual heavy-metal resistance (Huber et al., 1989; Peeples and Kelly, 1995; Auernik et al., 2008; McCarthy et al., 2014). This facultative chemolithotroph is capable of bioleaching, and the key to its chemical attack of metal ores is the redox regeneration of Fe^3+^ from Fe^2+^. Apart from Fe-oxidizing properties, metabolically versatile *M. sedula* has the ability to use a variety of electron donors, including reduced inorganic sulfur compounds, uranium ores, as well as molecular hydrogen under microaerobic conditions (Auernik and Kelly, 2008, 2010a; Maezato et al., 2012; Mukherjee et al., 2012; Wheaton et al., 2016).

In light of future perspectives of space exploration and *in situ* resource utilization (ISRU) programs, the possible implications of the extreme thermoacidophile *M. sedula* have been already suggested for asteroid biomining (Reed, 2015). A deeper investigation of the physiology of metal transforming microorganisms and mineral–microbial interactions facilitates our understanding of the possible energy production mechanisms of early life forms. Further, it extends our understanding of putative biosignatures that can be detected during missions aiming to uncover evidence of past habitability on planetary bodies.

The main goal of this work was to explore the growth potential and metal extraction capacity of the extremely thermoacidophilic archeon *M. sedula* cultivated on four different types of Mars regolith simulants – JSC Mars 1A, P-MRS, S-MRS, and MRS07/52 as the sole energy sources, as well as to investigate the possible biosignatures of mineral–microbial interactions associated with these cases. The different simulants were used to mimic the Martian regolith composition from different locations and historical periods of Mars: a palagonitic tephra (JSC Mars 1A as a close spectral analog to the bright regions of Mars); Early Hydrous or Phyllosilicatic Mars Regolith Simulant (P-MRS, characterized by high clay content); Late Acidic or Sulfatic Mars Regolith Simulant (S-MRS, characterized by the gypsum); the highly porous Mars Regolith Simulant (MRS07/52 that simulate sediments of the Martian surface). Due to its metal oxidizing metabolic activity, when given an access to these Martian regolith simulants, *M. sedula* released soluble metal ions into the leachate solution and altered their mineral surface leaving behind specific signatures of life.

## Materials and Methods

### Strain and Media Composition

*Metallosphaera sedula* (DSMZ 5348) cultures were grown aerobically in DSMZ88 Sulfolobus medium containing 1.3 g (NH_4_)_2_SO_4_, 0.28 g KH_2_PO_4_, 0.25 g MgSO_4_⋅7 H_2_O, 0.07 g CaCl_2_⋅2 H_2_O and 0.02 g FeCl_3_⋅6 H_2_O dissolved in 1 L of water. After autoclaving, Allen’s trace elements solution was added to 1 L media resulting in 1.80 mg MnCl_2_⋅4 H_2_O, 4.50 mg Na_2_B_4_O_7_⋅10 H_2_O, 0.22 mg ZnSO_4_⋅7 H_2_O, 0.05 mg CuCl_2_⋅2 H_2_O, 0.03 mg Na_2_MoO_4_⋅2 H_2_O, 0.03 mg VSO_4_⋅2 H_2_O, and 0.01 mg CoSO_4_ final concentration. The pH was adjusted to 2.0 with 10 N H_2_SO_4_. Chemicals of high purity grade were used for media preparation.

### Composition of Synthetic Martian Regolith Analogs

In this study, four mineral mixtures of Mars regolith simulants (MRS) were used to examine whether these minerals could provide nutrients/energy sources necessary for lithoautotrophic growth of *M. sedula* (**Tables 1, 2**). The mineral mixtures of synthetic Martian regolith analogs were assembled in accordance to data on the structural and chemical composition of Martian minerals identified in meteorites (McSween, 1994) and by recent orbiter and rover missions (Bibring et al., 2005; Poulet et al., 2005; Chevrier and Mathé, 2007; Mustard et al., 2009; Morris et al., 2010) reflecting current knowledge of environmental changes on Mars (Boettger et al., 2012). JSC Mars-1A Martian Regolith Simulant is a palagonitic tephra (volcanic ash altered at low temperatures), produced by Orbital Technologies Corporation, Madison, WI, United States. The only phases detected by x-ray diffraction are plagioclase feldspar and minor magnetite. Iron Mossbauer spectroscopy also detected traces of hematite, olivine, pyroxene and/or glass (Morris et al., 1993). One of the Mars regolith simulants was phyllosilicate-rich (Phyllosilicate Mars Simulant, P-MRS), containing a high percentage of smectite clays like montmorillonite, kaolinite, and chamosite, as well as carbonates (siderite, hydromagnesite). The other mixture (Sulfatic Mars Simulant, S-MRS) was characterized by its high gypsum and goethite content. Both mineral mixtures consist also of pyroxene and plagioclase (gabbro), olivine, quartz, and the anhydrous ferric oxide hematite (Gooding, 1978; Boettger et al., 2012). The mineral composition of P-MRS is modeled based on the phyllosilicate-rich sites on Mars, which formed during an aqueous weathering regime with neutral to alkaline conditions in the Noachian epoch (>3.7 Ga), either on the surface or in the subsurface at hydrothermal areas (**Tables 1, 2**) (Bibring et al., 2006; Chevrier and Mathé, 2007; Halevy et al., 2007; Bishop et al., 2008; Ehlmann et al., 2011). S-MRS represents the Martian sediments with a high sulfate content that presumably correspond to the acidic conditions in the Hesperian epoch (3.7–3.0 Ga) (Bibring et al., 2006; Bullock and Moore, 2007; Chevrier and Mathé, 2007). The final inorganic mineral mixture investigated in this study was the Martian soil simulate MRS07/52. This sample was supplied by German Aerospace Center and was produced to have comparable constituents to that of the soil on Mars (Moeller et al., 2008) (**Tables 1, 2**).

**Table 1:** Chemical composition of the synthetic Martian regolith analogs used in this study.

Major chemical composition	JSC Mars-1A^1^	P-MRS^2^	S-MRS^3^	MRS07/52^4^
	
	Wt %	Wt %	Wt %	Wt %
Silicon dioxide (SiO_2_)	34.5–44	43.6	30.6	34.6
Aluminum oxide (Al_2_O_3_)	18.5–23.5	11.9	9.2	14.1
Titanium dioxide (TiO_2_)	3–4	0.36	0.05	0.1
Ferric oxide (Fe_2_O_3_)	9–12	19.6	14.9	20.6
Iron oxide (FeO)	2.5–3.5	–	–	–
Magnesium oxide (MgO)	2.5–3.5	4.52	10.3	3.4
Calcium oxide (CaO)	5–6	4.74	17.8	6.1
Sodium oxide (Na_2_O)	2–2.5	0.32	1.09	2.5
Potassium oxide (K_2_O)	0.5–0.6	1.04	0.13	0.2
Manganese oxide (MnO)	0.2–0.3	0.16	0.3	–
Diphosphorus pentoxide (P_2_O_5_)	0.7–0.9	0.55	0.05	–
Sulfur trioxide (SO_3_)	–	0.2	9.1	5.1
LOI	–	11.8	5.4	–

*^1^Data for JSC Mars-1A obtained from Orbital Technologies Corporation, Madison, WI, United States; ^2,3^Data for P-MRS and S-MRS were obtained from Boettger et al. (2012); ^4^Data for MRS07/52 were obtained from Moeller et al. (2008).*

**Table 2:** Mineral composition of the synthetic Martian regolith analogs used in this study.

Mineral component	JSC 1A^1^	P-MRS^2^	S-MRS^3^	MRS07/52^4^
	
	Wt %	Wt %	Wt %	Wt %
Plagioclase Feldspar	64	–	–	–
Olivine	12	–	–	-
Magnetite	11	–	–	–
Pyroxene and/or glass	9	–	–	–
Gabbro		3	32	–
Dunite		2	15	–
Quartz		10	3	1
Hematite	5	5	13	20
Montmorillonite		45	–	48
Chamosite		20	–	–
Kaolinite		5	–	10
Siderite		5	–	–
Hydromagnesite		5	–	–
Goethite		–	7	–
Gypsum		–	30	–
Anhydrite		–	–	13
MgSO_4_		–	–	7
Halite		–	–	1

*^1^Data for JSC Mars-1A were obtained from Morris et al. (1993); ^2,3^Data for P-MRS and S-MRS were obtained from Boettger et al. (2012); ^4^Data for MRS07/52 were obtained from Moeller et al. (2008).*

### Cultivation Setup

Chemolithoautotrophic cultivation of *M. sedula* was performed in DSMZ88 Sulfolobus medium described above in 1 L glassblower modified Schott-bottle bioreactors (Duran DWK Life Sciences GmbH, Wertheim/Main, Germany), equipped with a thermocouple connected to a heating and magnetic stirring plate (IKA RCT Standard/IKA C-MAG HS10, Lab Logistics Group GmbH, Meckenheim, Germany) for temperature and agitation control. Each bioreactor was equipped with three 10 mL graduated glass pipettes, permitting carbon dioxide and air gassing (with the gas flow of 9 mL min^-1^, adjusted to five bubbles s^-1^ by using 8 mm valves (Serto, Frauenfeld, Switzerland)) and sampling of culture, respectively (Supplementary Figure 1). The graduated pipettes used for gassing were connected by silicon tubing to sterile 0.2 μm filters (Millex-FG Vent filter unit, Millipore, Billerica, MA, United States). The graduated pipettes used for sampling were equipped with a Luer-lock system in order to permit sampling with sterile syringes (Soft-Ject, Henke Sass Wolf, Tuttlingen, Germany). The offgas was forced to exit via a water-cooled condenser (Ochs GmbH, Bovenden, Germany). For the cultivations of *M. sedula* at 73°C the temperature inside the bioreactors was controlled by electronic thermocouple via the heating and magnetic stirring plates. *M. sedula* inocula were obtained by resuspending a chemolithoautotrophically grown cell pellet formed by centrifugation at 6000 × *g* for 15 min in DSMZ88 media without organic carbon source and inorganic metals/metalloids as energy sources. For chemolithoautotrophic growth cultures with initial pH of 2.0 were supplemented with 1 g/liter Martian regolith simulants, no further pH adjustments were introduced during the cultivation. The minerals were temperature sterilized at 180°C in a heating chamber for a minimum of 24 h prior to autoclaving at 121°C for 20 min. Abiotic controls consisting of uninoculated culture media supplemented with MRSs were included in all the experiments. Growth of cells was monitored by phase contrast/epifluorescence microscopy and metal release. Precise cell enumeration was found to be difficult due to the interference with mineral particles of similar size and round shaped morphology and shielding of *M. sedula* by iron mineral precipitates, especially at later stages of the growth. To visualize wiggling cells on solid particles, a modified “DAPI” (4′-6′- Diamidino-2-phenylindole) staining was used (Huber et al., 1985); afterward the cells were observed and recorded with ProgRes^®^ MF cool camera (Jenoptik) mounted on Nikon eclipse 50i microscope, equipped with F36-500 Bandpass Filterset (ex, 377/50 nm; em, 447/60 nm).

### Multi-Labeled-Fluorescence *In Situ* Hybridization (MiL-FISH)

Cultures of *M. sedula* grown on Martian Regolith Simulants (Supplementary Figure 2) were fixed in 2% paraformaldehyde (PFA) at room temperature for 1 h and subsequently washed three times in 1x PBS (phosphate buffer saline) with centrifugation steps of 3000 × *g* for 5 min between each exchange. Cells were extracted from sediment after Braun et al. (2016) as follows: 1 ml sediment from each sample was centrifuged at 5000 × *g* for 5 min, the supernatant discarded, re-suspended in 1.5 ml Mili-Q that included 0.2 ml methanol and 0.2 ml detergent mix (100 mM EDTA, 100 mM sodium pyrophosphate decahydrate and 1% v:v Tween 80) and shaken at 750 rpm for 60 min. To separate cells from sediment particles samples were sonicated at 30% power three times for 15 s using an ultrasonic probe. Finally, a gradient centrifugation was applied consisting of three 2 ml Nycodenz layers of 30, 50, and 80% on top of a 2 ml sodium polytungstate solution with a 2.23 g per ml density and centrifuged at 5000 × *g* for 2 h at 4°C. The microbial fraction contained within the supernatant above the sodium polytungstate was extracted with a glass pipette. To further clean the samples from fine sediment particles the gradient centrifugation was repeated a second time in the same manner. *M. sedula* 16S rRNA phylotype specific probe was designed with the software package ARB (Ludwig et al., 2004) and labeled with 4x Atto488 via Click chemistry (biomers.net GmbH, Ulm, Germany) (see **Table 3**). Cells were mounted on 10 well Diagnostica glass slides (Thermo Fisher Scientific Inc. Waltham, MA, United States) and MiL-FISH conducted directly on slides with 30% formamide and a 3 h hybridization time (Schimak et al., 2015). Positive control for the specificity of the phylotype specific probe M.sedula_174 was provided by use of the same *M. sedula* DSM5348 culture published in Schimak et al. (2015). After hybridization, slides were washed for 15 min at 48°C according to Manz et al. (1992) (14 to 900 mM NaCl, 20 mM Tris-HCl [pH 8], 5 mM EDTA [pH 8], and 0.01% SDS) at a stringency adjusted to the formamide concentration used (Manz et al., 1992). DNA staining with DAPI (4 = ,6-diamidino-2-phenylindole) followed (10 mg/ml) for 10 min after which slides were rinsed in distilled water three times. Vectashield (Vector Laboratories, Burlingame, CA, United States) mounting medium was applied and slides closed with a coverslip. Fluorescence images were taken with an AxioCam Mrm camera mounted on an Nikon Eclipse 50i illuminated by a Nikon Intensilight C-HGFI light source and equipped with a F36-525 Alexa 488 (ex, 472/30 nm; em, 520/35 nm) filter cube. Images were recorded with the Windows based AxioVision (release 4.6.3 SP1) imaging software and any image-level adjustments made either therein or using the Mac OS X based Adobe Photoshop version 12.0.4. The figure table depicted was composed using Mac OS X based Adobe Illustrator version 15.0.2 and images cropped by use of clipping masks.

**Table 3:** Oligonucleotide probes used in this study.

Probe	Sequence 5′–3′ (reverse complementary)	Target gene	Label	Synthesis	Taxon	Target species	FA %	Colour
M. sedula_174_17mer	AGA UUC CCU UGC CCG CU	16S rRNA	Atto488	Click chemistry	Archaea	*Metallosphaera sedula*	30%	Green

*Probe name, nucleotide sequence - underscore indicates nucleotide:fluorochrome conjugates, target gene, label type, label synthesis, target taxon or higher, target species and probe color during imaging.*

### Scanning Electron Microscopy

The mineral precipitates (Supplementary Figure 3) were examined with a Zeiss Supra 55 VP scanning electron microscope (SEM), equipped with a spectroscope of dispersive energy (EDS), which was used for imaging and elemental analysis of precipitates. The samples were coated with a thin Au/Pd layer (Laurell WS-650-23 spincoater). The acceleration voltage applied was 5 kV and the EDS analyses were performed with a 120 μm aperture and a counting time of 50 s. In order to control the beam parameters, cobalt was used as a standard. Conventional ZAF matrix correction was used to calculate the final composition from the measured X-ray intensities. All the sample spots investigated by EDS were chosen randomly and each spot was measured three times. **Table 4** represents the chemical composition of aluminum/chlorine containing microspheroids, which was taken as the average of the measurements from 20 randomly chosen spots.

**Table 4:** Average metal composition (%) of microhemispheroids detected in mineral precipitates withdrawn from cultures of *Metallosphaera sedula* grown on JSC Mars 1A, P-MRS, and S-MRS.

	C	O	Al	Cl
Mean % (± standard deviation) *n = 20*	6.41 ± 5.01	20.33 ± 7.71	18.18 ± 10.47	7.10 ± 3.85

### Metal Analysis

To determine the extracellular concentrations of metal ions mobilized from the Martian regolith simulants, culture samples were clarified by centrifugation. Samples of the resulting supernatants were filtered (0.44 μm pore size) and analyzed by inductively coupled plasma-optical emission spectrometer (ICP-OES) Perkin Elmer Optima 5300 DV. All reported values are averages from duplicate samples.

### Electron Paramagnetic Resonance (EPR)

The Electron Paramagnetic Resonance (EPR) spectra were recorded on an X-Band Bruker Elexsys-II E500 CW-EPR spectrometer (Bruker Biospin GmbH, Rheinstetten, Germany) at 90 ± 1 and 293 ± 1 K using a high sensitivity cavity (SHQE1119). Solid state EPR measurements were performed setting microwave frequency to 9 GHz, modulation frequency to 100 kHz, center field to 6000 G, sweep width to 12000 G, sweep time to 335.5 s, modulation amplitude to 20.37 G, microwave power to 15 mW, conversion time to 81.92 ms and resolution to 4096 points. The samples were put in EPR quartz tubes (Wilmad-LabGlass, Vineland, NJ, United States) and scanned three times, of which the average was used for analysis. The spectrum of an empty control tube was subtracted from all sample spectra. All spectra were analyzed with the Bruker Xepr software.

### Statistical Analysis

For the statistical analysis and graphical representation of the data the Excel 2016 (version 7.0) and Sigma plot (version 13.0) software packages were used.

## Results

### Chemolithotrophic Growth on Synthetic Martian Regolith

**FIGURE 1:**
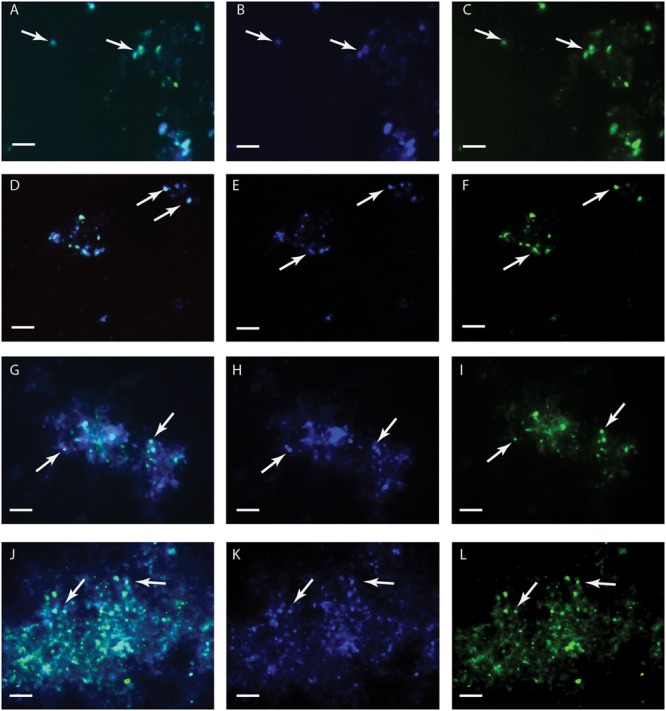
Multi-Labeled-Fluorescence *in situ* Hybridization (MiL-FISH) of *Metallosphaera sedula* cells grown on synthetic Martian regolith materials as the sole energy sources. **(A,D,G,J)** Overlaid epifluorescence images, showing overlap of the specific oligonucleotide probe targeting *M. sedula* with DAPI signals. **(B,E,H,K)** DAPI staining of the same field (blue). **(C,F,I,L)** MiL-FISH images of cells (green) after hybridization with the specific oligonucleotide probe targeting *M. sedula*. Cultures of *M. sedula* were examined with MiL-FISH conducted after Schimak et al. (2015) after 21 days of cultivation with JSC Mars 1A **(A–C)**, P-MRS **(D–F)**, S-MRS **(G–I)**, and MRS07/52 **(J–L)**.

*Metallosphaera sedula* was grown under chemolithoautotrophic conditions on MRSs (**Table 5**), and interaction of cells with the mineral particles was examined. After 21 days of CO_2_-supplemented cultivation on MRSs as the sole energy source, phylogenetic identification and visualization of *M. sedula* cells was achieved with Multi-Labeled fluorescence *in situ* hybridization (MiL-FISH). For this purpose a newly designed phylotype specific probe targeting the 16S rRNA (**Table 3**) was used. The cultures of *M. sedula* grown on all four investigated in this study MRSs (JSA 1A, P-MRS, S-MRS, and MRS07/52) resulted in positive fluorescent signal with Atto488 labeled probes at a 30% formamide concentration (**Figure 1**). Only cells that show both DAPI and fluorescent signal were considered as positive hybridization with the target organism. Additionally, it was noted that cells of S-MRS and MRS07/52 cultures occur in mucus bound aggregates which can be inferred as extracellular polysaccharide substances (EPS) known for *M. sedula* (Auernik et al., 2008) (**Figures 1G–L**).

**Table 5:** Cell densities of *M. sedula* cultures grown on the synthetic Martian regolith analogs at “0” time point and after 21 days of cultivation.

Cultivation time	0 day	21 days
	
	Cell density [cells/ml]	
JSC 1A	4,93E+06 ± 6,49E+05	1,24E+08 ± 7,40E+07
P-MRS	5,98E+06 ± 3,19E+06	4,77E+08 ± 3,74E+08
S-MRS	5,57E+06 ± 3,10E+06	3,06E+08 ± 3,07E+08
MRS07/52	4,34E+06 ± 2,25E+06	2,02E+08 ± 1,32E+08
Pyrite	9,80E+06 ± 9,45E+05	1,50E+09 ± 2,50E+08
No MRSs added	6,95E+06 ± 7,85E+05	1,48E+05 ± 8,73E+03

*Mean and standard deviation of *n* = 3 biological replicates are represented. When no MRSs were added, cultivation medium was inoculated with cells of *M. sedula* without additional energy source.*

### Metal Release

**FIGURE 2:**
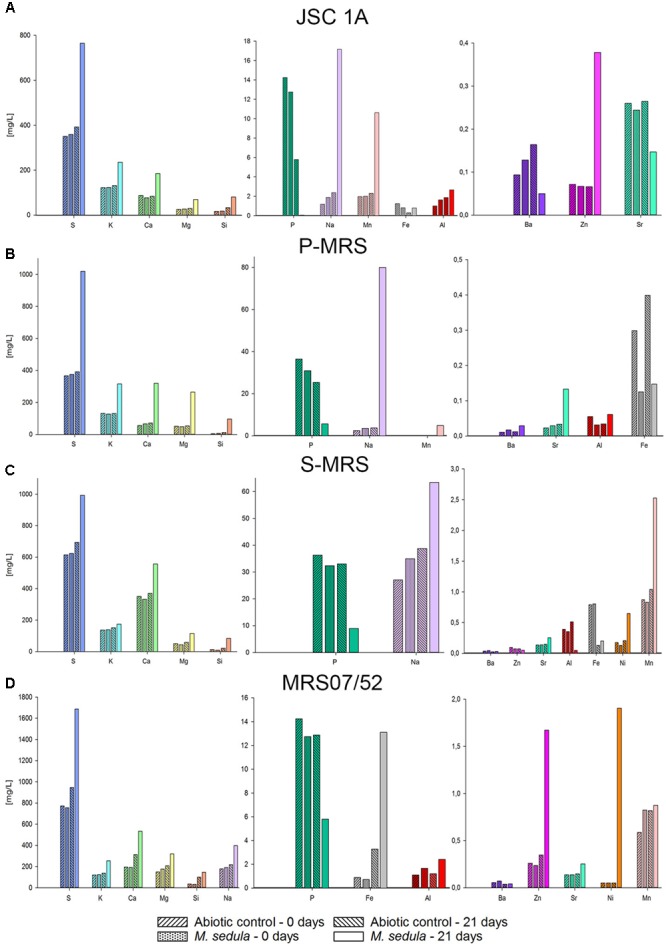
Inductively coupled plasma-optical emission spectrometer (ICP-OES) analysis of released metal ions in supernatant of *M. sedula* cultures grown on the Martian regolith simulants [JSC Mars 1A **(A)**; P-MRS **(B)**; S-MRS **(C)**; MRS07/52 **(D)**] as the sole energy sources. Samples were taken at “0” time point and after 21 days of cultivation of *M. sedula* on the Martian regolith simulants and from corresponding abiotic controls.

Inductively coupled plasma-optical emission spectrometer analysis of composition of major elements mobilized from all tested Martian regolith simulants showed the elevated levels of released S, K, Ca, Mg, Si, and Na in the growth medium (leachate solution) (**Figure 2**). A further change in trace elements (higher released Mn) occurred in cultures of *M. sedula* grown on JSA 1A, P-MRS, and S-MRS. Additionally, in case with MRS07/52 leachate solution was characterized by elevated levels of Fe, Ni, and to lesser extent Al in comparison to abiotic control (**Figure 2D**). The increased level of released Ni ions was also detected in S-MRS grown cultures. The elevated levels of Sr ions were measured in culture supernatants of *M. sedula* grown on P-MRS, S-MRS, and MRS07/52, while increased soluble Zn was represented in JSC 1A and MRS07/52 grown cultures. The drop in P concentrations was detected in leachate solutions of all the tested MRS. The decrease of total iron observed in *M. sedula* cultures grown on JSC 1A, P-MRS, and S-MRS could be possibly attributed to the formation of insoluble iron oxyhydroxides which form precipitated mineral phase and therefore are not included in the samples of leachate solution used for the ICP-OES analysis. This phenomenon of ‘lost iron’ has been already previously reported in case of microbial cultivation on extraterrestrial material (Gronstal et al., 2009).

### SEM-EDS Investigation of Solid Mineral Phase

**FIGURE 3:**
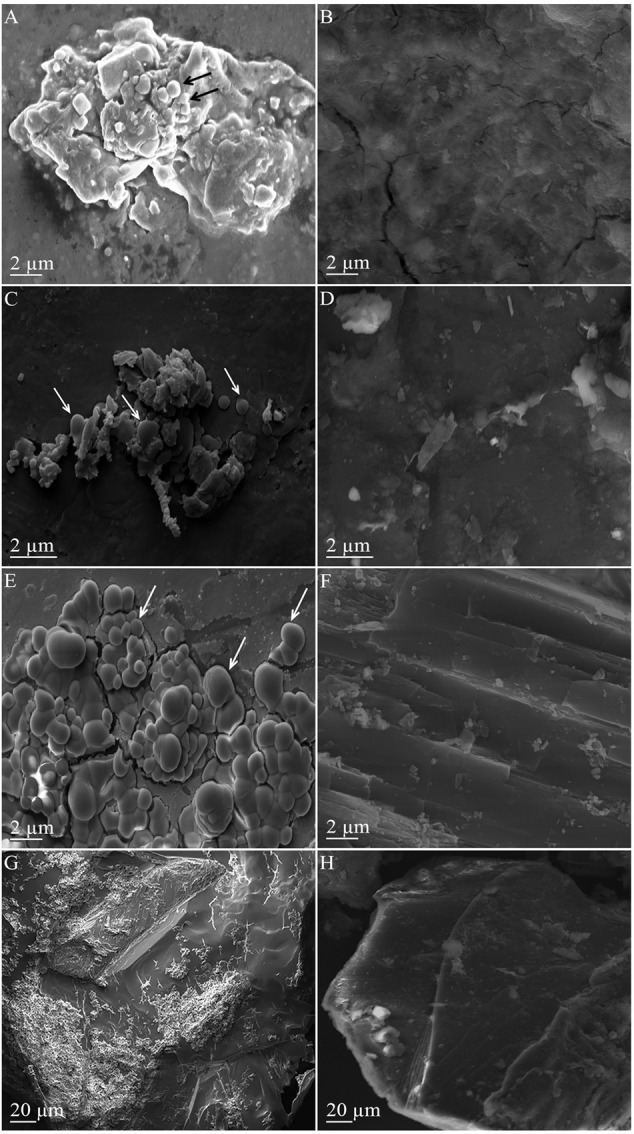
Scanning electron microscopy (SEM) images of the mineral surfaces of synthetic Martian regolith materials. **(A)** Scanning electron image showing a surface of mineral precipitate obtained after the cultivation of *M. sedula* on JSC 1A. **(C)** Scanning electron image showing a surface of mineral precipitate obtained after the cultivation of *M. sedula* on P-MRS. **(E)** Scanning electron image showing a surface of mineral precipitate obtained after the cultivation of *M. sedula* on S-MRS. **(G)** Scanning electron image showing a surface of mineral precipitate obtained after the cultivation of *M. sedula* on JSC 1A. Cultures of *M. sedula* were examined with SEM after 21 days of cultivation on the Martian regolith simulants. Aluminum/chlorine containing microspheroids occurred in mineral precipitates of JSC Mars 1A, P-MRS, and S-MRS after *M. sedula* growth, are depicted with arrows. Images **(B,D,F,H)** represent the corresponding abiotic controls.

To further study the interactions of *M. sedula* with the Martian regolith simulants, the surface of these minerals was examined for possible alterations upon *M. sedula* growth. Inspection by SEM of solid mineral phase in cultures of *M. sedula* grown on synthetic Martian regolith revealed the presence of sphere-like particles of variable size (0.3–3 μm). These microhemispheroids were represented in cultures of *M. sedula* grown on JSC 1A, P-MRS, and S-MRS and absent in the corresponding abiotic controls (**Figure 3**). The results of SEM-EDS analysis indicate that these hemispheroidal morphologies are mainly composed of oxygen, aluminum and chlorine and with no or low carbon content (**Table 4** and Supplementary Figure 4). The solid mineral phase withdrawn from S-MRS grown cultures of *M. sedula* was especially enriched with aluminum/chlorine containing microspheroids, while both biotransformed JSC 1A and P-MRS had a minor occurrence of these particles. **Figure 3C** and Supplementary Figure 4 show budding microspheroids represented on biotransformed surface of S-MRS. SEM assisted investigations of a solid phase of biotransformed MRS07/52 showed the biofilm layer distributed over the mineral surface (**Figure 3G**). Such a deposited biofilm layer was absent in the corresponding abiotic control of MRS07/52 incubated in the growth medium at 73°C but without *M. sedula* (**Figure 3H**).

### EPR Investigation of Solid Mineral Phase

**FIGURE 4:**
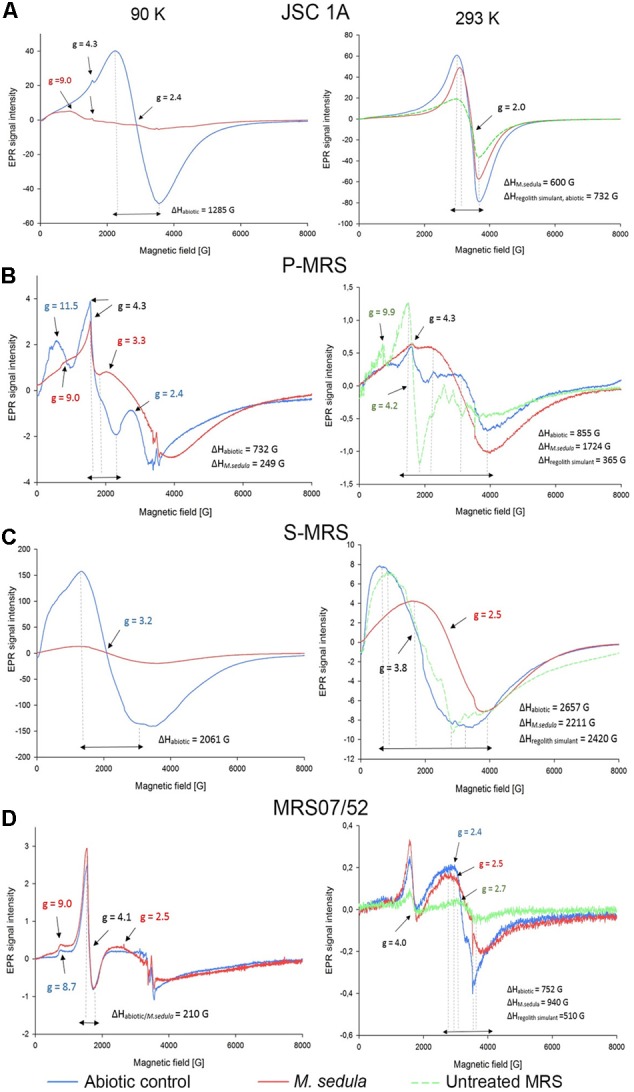
Electron Paramagnetic Resonance (EPR) spectra of raw synthetic Martian regolith materials (green line), synthetic Martian regolith materials bioprocessed by *M. sedula* (red line), and synthetic Martian regolith materials after the treatment with cultivation medium, but without *M. sedula* (abiotic control, blue line). **(A)** EPR spectra of JSC Mars 1A; **(B)** EPR spectra of P-MRS; **(C)** EPR spectra of S-MRS; **(D)** EPR spectra of MRS07/52). Spectra recorded at 90 and 293 K are represented in left and right columns, respectively. *g*- values are depicted with the color code corresponding to the spectra; identical *g*-values are represented in black color. The EPR *g*-values recorded at 90 and 293 K are grouped in Supplementary Table 1. The corresponding EPR Linewidths (ΔH) values are provided in Supplementary Table 2.

Electron Paramagnetic Resonance measurements were performed to (1) identify paramagnetic species of manganese and iron in the different MRSs samples and to (2) investigate the impact of *M. sedula* on MRSs with a possible effect on the oxidative state of manganese and iron. The four different MRSs mineral mixtures were incubated in cultivation medium in the presence (biotic) or absence (abiotic) of *M. sedula*. The spectra of untreated (raw) MRSs were investigated as well. The abiotic samples of JSC 1A and P-MRS contained Mn^2+^ as evident by a prominent signal at *g* = 2.4 with a broad linewidth of >1200 G (**Figure 4** and Supplementary Tables 1, 2) typical for abiotic Mn^2+^ samples (Kim et al., 2011; Ivarsson et al., 2015). For JSC 1A, the biotic sample revealed an almost complete decline of the Mn^2+^ signal, while for P-MRS an alteration of the *g* = 2.4 signal in the biotic sample resulted to the appearance of a broad signal with *g* = 3.3, indicating dipolar interactions of mixed ionic states (Mn^3+^ and Mn^4+^) (Ivarsson et al., 2015). In biotic and abiotic samples of JSC 1A and P-MRS, Fe^3+^ could be identified by the characteristic *g* = 4.3 and *g* = 9 signals. A high spin d5 configuration of the Fe^3+^ can be inferred from the positions of these resonance signals. **Figure 4B** shows that the amplitude of high spin Fe^3+^ *g* = 4.3 signal is slightly lower in biotic P-MRS than in the corresponding abiotic sample, while the signals *g* = 11.5 and *g* = 9.9 measured at 90 and 293 K, respectively, are clearly diminished after *M. sedula* cultivation. Independent of the incubation conditions, the EPR spectra of all four MRSs samples recorded at 90 K revealed small signals with *g*-values in the range of 2.0 and 2.7, which were assigned to Fe^3+^ with low spin electron configuration. No significant changes were detected between the biotic and abiotic samples measured at 90 K with regard to the Fe^3+^ in the low spin state. In opposite, a continuously narrowing resonance signal *g* = 2 was recorded at 293 K in JSC 1A samples after cultivation with *M. sedula*. The linewidth (ΔH) remained unaltered for raw and abiotic JSC 1A (732 G) and decreased to 600 G after the cultivation with *M. sedula* (**Figure 4** and Supplementary Table 2).

The EPR spectrum of the abiotic S-MRS sample exhibits a prominent resonance at *g* = 3.2 with a broad linewidth of 2061 G, which is absent in the EPR spectra of all the other MRSs samples measured at 90 K. This specific resonance is obtained from samples containing tetrahedral Mn^2+^ species (Xu et al., 1999). A pronounced decrease of the *g* = 3.2 resonance signal could be demonstrated for the biotic sample of S-MRS compared to the corresponding abiotic sample. In MRS07/52, Fe^3+^ species in the low and high spin state were identified based on the characteristic EPR signals as mentioned above. No manganese could be detected in MRS07/52. The minor difference in biotic and abiotic MRS07/52 samples occurred at the area of low spin Fe^3+^, which is even more detectable in spectra recorded at 293 K (**Figure 4D**). The slightly increased amplitude of high spin Fe^3+^ signal along with the shift of *g*-value from 8.7 for abiotic to 9 for biotic samples was also observed at 90 K. The strong resonance line at *g* = 4.1 which can be assigned to Fe^3+^ located in orthorhombic positions (Polikreti and Maniatis, 2002) is not altered in biotic and abiotic MRS07/52 samples (**Figure 4D**).

## Discussion

Evolutionally diversified metal-solubilizing microorganisms with their fascinating metabolic pathways have developed an exquisite set of capabilities for manipulating minerals by dissolving them to access useful metals. We tested the ability of *M. sedula* to grow chemolithoautotrophically in four different types of synthetic Martian regolith. As growth was detected in all four MRS, we concluded that *M. sedula* is capable of chemolithotrophic growth using the synthetic Martian regolith as the sole energy source. Further, our results indicated that *M. sedula* is able to solubilize metals from the synthetic Martian regolith (e.g., S, K, Ca, Na, Mg, Si, Mn, Fe, Zn, Ni, and Sr) into the growth medium (leachate solution). Interestingly, in all four tested MRSs, the amount of detected phosphorus decreased in the biogenic samples after 21 days of cultivation, possibly indicating phosphorous consumption by *M. sedula* in order to maintain the growing population of the cells, which can explain a decreased amount of P in leachate solutions (**Figure 2**). The observed metabolic activity coupled to the release of free soluble metals from the synthetic Martian regolith can certainly pave the way to asteroid biomining, launching the biologically assisted exploitation of raw materials from asteroids, meteors and other celestial bodies.

However, insufficient evidence exists to confidently identify which metal/or metals in all tested MRSs serve as electron donors utilized by *M. sedula* to satisfy its bioenergetics needs. Due to its versatile metal-oxidizing capacities (Huber et al., 1989; Auernik and Kelly, 2010a,b; Maezato et al., 2012; Mukherjee et al., 2012; Wheaton et al., 2016), the fact that *M. sedula* can potentially respire on a combination of different elements represented in MRSs cannot be ruled out. It would seem beneficial for *M. sedula* to attach preferentially to those minerals containing useable substrates such as reduced iron or sulfur compounds. Fe^2+^ as a constitutive element is represented in variety of minerals, including olivine [(Mg, Fe^2+^)_2_SiO_4_] (11% in JSC 1A, 2% in P-MRS, 15% in S-MRS), and siderite [Fe(CO_3_), 5% in P-MRS], or bound to the smectite clay chamosite [(Fe^2+,^Mg)_5_Al_2_Si_3_O_10_(OH)_8_, 20% in P-MRS] (**Table 2**). The comparison of the obtained EPR spectra of raw and abiotic samples (**Figure 4**, green and blue lines, correspondingly) with the mineralogy and chemical composition of all MRSs we tested suggested that the recorded signals are most likely due to Mn^2+^ and Fe^3+^ ions. The obvious alteration of the width of EPR signals *g* = 2 detected in JSC 1A samples at 293 K after *M. sedula* growth might well reflect the oxidation of Fe^2+^ into Fe^3+^ (Presciutti et al., 2005; Mangueira et al., 2011). Interestingly, no Δ*H* alteration of *g* = 2 signal was observed for abiotically treated JSC 1A compared to raw JSC 1A material (**Figure 4A** and Supplementary Table 2). The slight increase of the signal amplitude of high spin Fe^3+^ *g* = 4.1 and *g* = 9 signals along with the appearance of *g* = 2.5 signal in biotic samples of MRS07/52 suggest the accumulation of Fe^3+^ in mineral phase, which also speaks on the account of Fe^2+^ oxidation mediated by *M. sedula* (**Figure 4D**). Interestingly, MRS07/52 is the only simulant among all tested MRSs, where Fe ions were detected to be released from after 21 days of cultivation with *M. sedula*. In biotic samples of Mn-bearing MRSs (JSC 1A, P-MRS, and S-MRS) the increased signals of mixed ionic states Mn^3+^ and Mn^4+^ and the decrease of Mn^2+^ in tetrahedral positions were observed pointing to a plausible redox couple for *M. sedula* respiration. The accumulation of redox heterogeneous Mn species may occur in the mineral phase due to *M. sedulas* oxidative metabolism, analogously to microbial mediated sulfur oxidation with the wide variety of redox heterogeneous intermediate sulfur compounds (Schippers et al., 1996) and serve as a fingerprint of chemolithoautotrophic life. However, to which extent Mn^2+^ is oxidized to Mn^3+^ and Mn^4+^ by *M. sedula* cannot be elucidated by EPR.

The signal g≅4 assigned for high spin energy Fe^3+^ is well preserved within JSC 1A, P-MRS, and MRS07/52 abiotic and biotic samples, with only small variations in amplitude. This situation indicates that the Fe^3+^ remained associated to orthorhombic positions in the structure of mineral precipices upon *M. sedula* growth. Such an observation is also in line with EPR-characterization of iron-bearing multi-mineral materials under the oxidative conditions of firing temperatures, suggesting that a biologically mediated oxidative effect does not differ from abiotic physical–chemical oxidation in this spectral area. However, changes were detected at signals *g* = 9 (**Figures 4A,B,D** and Supplementary Table 1) originating from high-spin rhombic Fe^3+^ centers, supporting the microbial mediated alteration of Fe^3+^ complexes in axial symmetry.

The different composition of mineral phases of MRSs has to be taken into account when assigning a certain resonance signal and the formation of different environments in response to abiotic and biologically mediated oxidation. Nevertheless, the EPR data should be supported by other observations such as synchrotron assisted X-ray absorption techniques, to obtain a deeper insight into the microbial mediated mineralogical alterations.

The development of the microspheroids described here that overgrow mineral surface of Martian regolith simulants was one of the features mediated by *M. sedula*. Most of the microspheroids were observed in the range of 0,3 to 3 μm in size, with the majority in between 0,5 and 1 μm. Frequently, the microspheroids were characterized by very low carbon content, which did not exceed the background carbon level in surrounding mineral surface according to our EDS analysis and suggested the non-cellular nature of these morphologies (Supplementary Figure 4). Chlorine and aluminum content of the microspheroids has been constantly detected by EDS analysis, with chlorine represented solely in microspheroid structures and absent in the mineral background (Supplementary Figure 4 and **Table 4**). These microspheroid structures tend to cluster into aggregates, which overgrow the mineral surfaces while forming assemblages on underlying structures (**Figure 3E** and Supplementary Figure 4).

Occurring as single particles (**Figures 3A,B**), these microstructures expose correctly shaped hemispherical surface. When grouped into aggregates, overgrowing the surface of S-MRS and competing with each other in restricted size area, microspheroids expose overlapping conjoined boundary sides, which results in a distorted hemispherical morphology and “overcrowded” appearance (**Figure 3C**). The neoformed opaline microhemispheroids of similar morphology were previously described as a part of possibly microbial mediated diagenesis of marine sediments (Monty et al., 1991). Massively deposited chlorine/aluminum microspheroids can be inferred to be self-assembled aggregated clusters that form new nuclei and mineral intermediates as a part of new mineral formation process biologically mediated by *M. sedula*. This fine-scale morphological signature along with leaching of elements as the signs of metabolic activity may serve as indication of chemolithotrophic life in extreme environments.

## Conclusion

The mineralogical composition of the synthetic Martian regolith analogs supports the chemolithotrophic growth of *M. sedula*, when Martian regolith simulants are used as the sole energy sources. Acquisition of Fe^2+^ and/or Mn^2+^ from these simulants seems to satisfy the bioenergetic needs of *M. sedula*. The obtained results highlight metallophilic life in extreme environments and reveal unique fingerprints of life in the extreme conditions.

## Author Contributions

DK, MS, MP, OS, AB, and TM performed experiments. DK and MP planned, performed, and interpreted EPR experiments; all authors provided editorial input. All authors made substantial contributions to the acquisition, analysis, and interpretation of data described in this perspective. All authors critically reviewed the report and approved the final version.

## Conflict of Interest Statement

The authors declare that the research was conducted in the absence of any commercial or financial relationships that could be construed as a potential conflict of interest.
