# Supplementary material for: Exploring Fingerprints of the Extreme Thermoacidophile Metallosphaera sedula Grown on Synthetic Martian Regolith Materials as the Sole Energy Sources
Source: Front Microbiol. 2017 Oct 9;8:1918. doi: 10.3389/fmicb.2017.01918 (PMC5640722; doi:10.3389/fmicb.2017.01918)
Supplement: Supplementary file 1 [file Table_1.DOCX]

| **Temperature** | **Condition** | **JSC 1A** | **P-MRS** | **S-MRS** | **MRS07/52** |
| --- | --- | --- | --- | --- | --- |
| **293 K** | Untreated regolith simulant | 2.0 | 4.2 9.9 | 3.8 | 2.7 4.0 |
|  | Abiotic control | 2.0 | 4.3 | 3.8 | 2.4 4.0 |
|  | *M. sedula* cultivation | 2.0 | 4.3 | 2.5 | 2.5 4.0 |
| **90 K** | Abiotic control | 4.3 2.4 | 4.3 11.5 2.4 | 3.2 | 8.7 4.1 |
|  | *M. sedula* cultivation | 4.3 2.4 9.0 | 4.3 9.0 3.3 | 3.2 | 9.0 4.1 2.5 |

**Supplementary Table 1. EPR g-values recorded at 90 K and 293 K for Martian Regolith Simulants JSC 1A, P-MRS, S-MRS, and MRS07/52 before and after cultivation with *M. sedula*.**
