# Supplementary material for: Exploring Fingerprints of the Extreme Thermoacidophile Metallosphaera sedula Grown on Synthetic Martian Regolith Materials as the Sole Energy Sources
Source: Front Microbiol. 2017 Oct 9;8:1918. doi: 10.3389/fmicb.2017.01918 (PMC5640722; doi:10.3389/fmicb.2017.01918)
Supplement: Supplementary file 2 [file Table_2.DOCX]

| **Temperature** | **Condition** | **JSC 1A** | **P-MRS** | **S-MRS** | **MRS07/52** |
| --- | --- | --- | --- | --- | --- |
| **293 K** | Untreated regolith simulant | 732 | 365 | 242 | 510 |
|  | Abiotic control | 732 | 855 | 2657 | 752 |
|  | *M.sedula* cultivation | 600 | 1724 | 2211 | 940 |
| **90 K** | Abiotic control | 1285 | 732 | 2061 | 210 |
|  | *M. sedula* cultivation | - | 249 | - | 210 |

**Supplementary Table 2. EPR Linewidths (ΔH) for Martian regolith simulants [G] recorded at 77 K and 293 K before and after cultivation with *M. sedula*.**
