# Supplementary figures and images for: Exploring Fingerprints of the Extreme Thermoacidophile Metallosphaera sedula Grown on Synthetic Martian Regolith Materials as the Sole Energy Sources
Source: Front Microbiol. 2017 Oct 9;8:1918. doi: 10.3389/fmicb.2017.01918 (PMC5640722; doi:10.3389/fmicb.2017.01918)

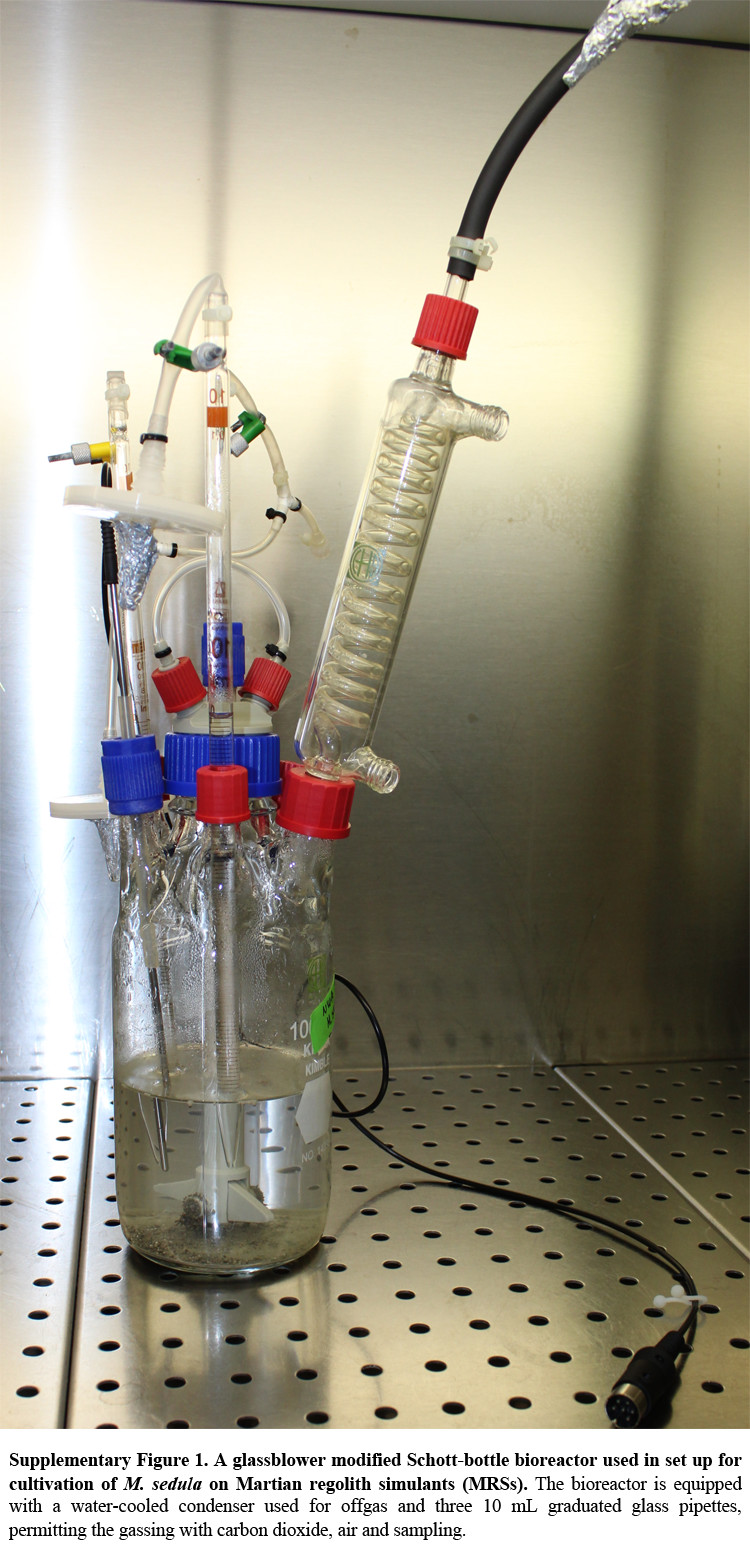

Supplement: Supplementary file 3 [file Image_1.JPEG]

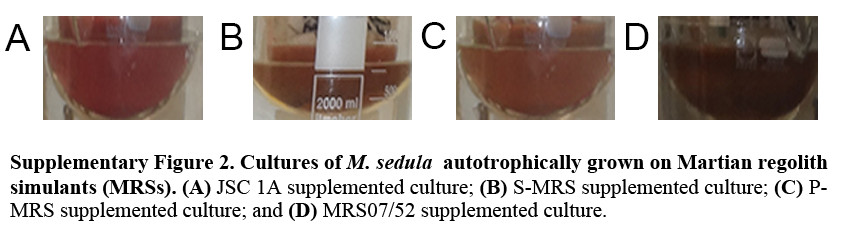

Supplement: Supplementary file 4 [file Image_2.JPEG]

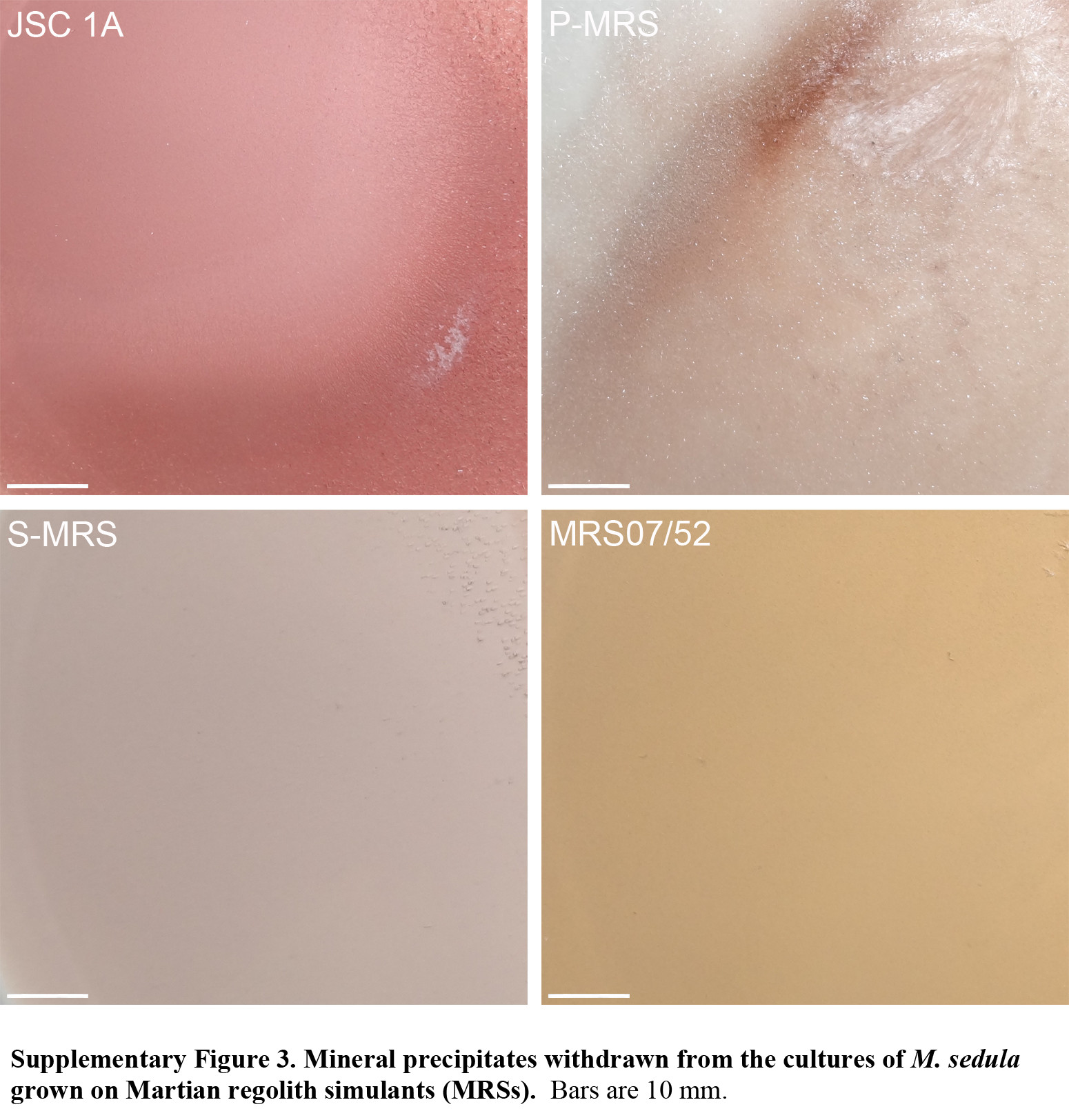

Supplement: Supplementary file 5 [file Image_3.JPEG]

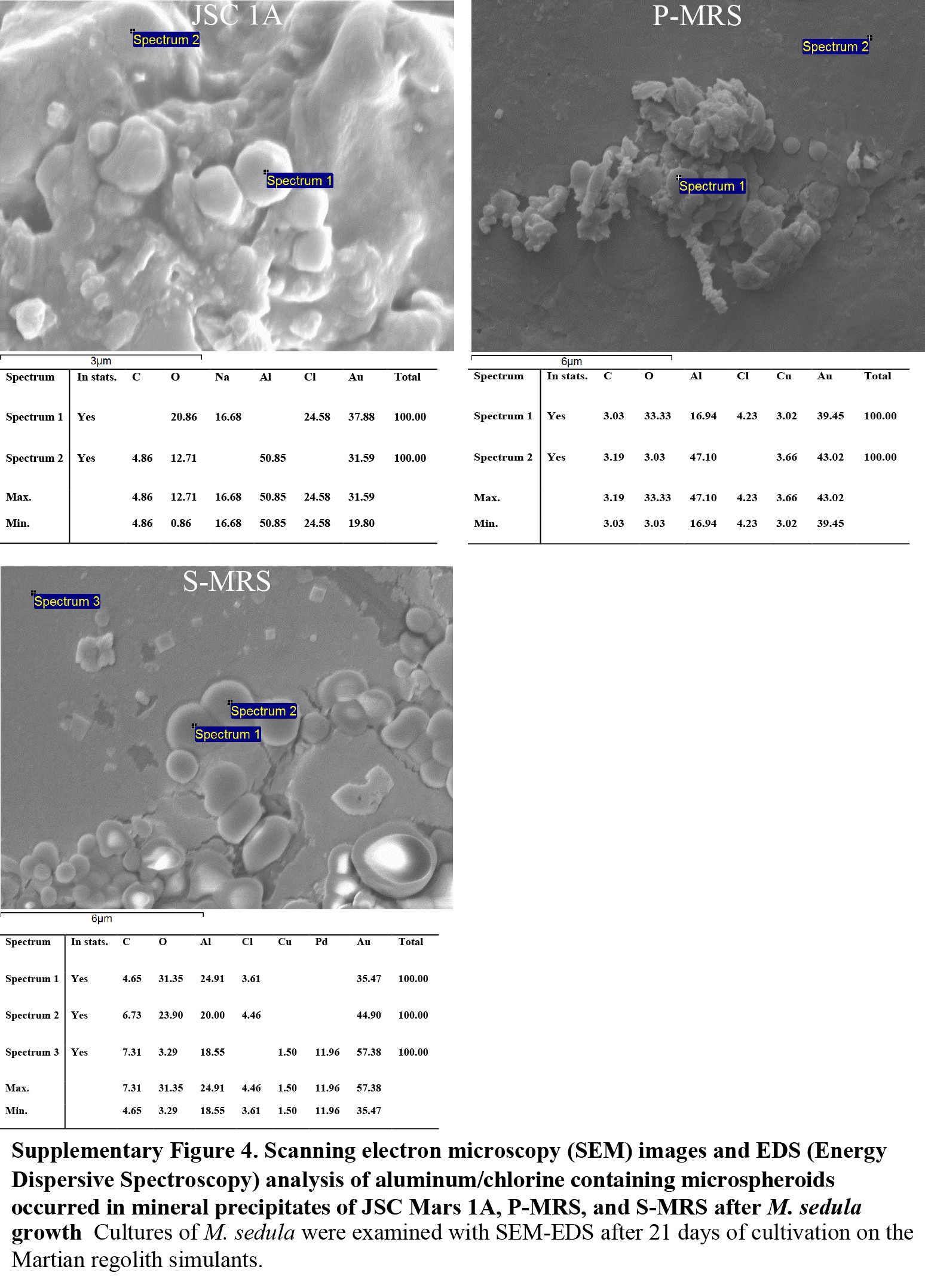

Supplement: Supplementary file 6 [file Image_4.JPEG]
